# Review on the methodology to assess respiratory tract lesions in pigs and their production impact

**DOI:** 10.1186/s13567-023-01136-2

**Published:** 2023-02-01

**Authors:** Dominiek Maes, Marina Sibila, Maria Pieters, Freddy Haesebrouck, Joaquim Segalés, Luís Guilherme de Oliveira

**Affiliations:** 1 Faculty of Veterinary Medicine, Department of Internal Medicine, Reproduction and Population Medicine, Unit of Porcine Health Management, Salisburylaan 133, 9820 Merelbeke, Belgium; 2 Unitat Mixta d’Investigació IRTA-UAB en Sanitat Animal. Centre de Recerca en Sanitat Animal (CReSA), Campus de La Universitat Autònoma de Barcelona (UAB), Bellaterra, 08193 Catalonia, Spain; 3 IRTA. Programa de Sanitat Animal. Centre de Recerca en Sanitat Animal (CReSA), Campus de la Universitat Autònoma de Barcelona (UAB), Bellaterra, 08193 Catalonia, Spain; 4 OIE Collaborating Centre for the Research and Control of Emerging and Re-Emerging Swine Diseases in Europe (IRTA-CReSA), 08193 Barcelona, Bellaterra Spain; 5 Department of Veterinary Population Medicine, Veterinary Diagnostic Laboratory & Swine Disease Eradication Center, College of Veterinary Medicine, University of Minnesota, St. Paul, MN USA; 6 Departament de Sanitat i Anatomia Animals, Facultat de Veterinària, Universitat Autònoma de Barcelona, Bellaterra, 08193 Barcelona, Spain; 7 School of Agricultural and Veterinarian Sciences, São Paulo State University (Unesp), Jaboticabal, Brazil

**Keywords:** Pigs, lung, lesion, slaughterhouse, cranioventral pulmonary consolidation, pleurisy, impact

## Abstract

Porcine respiratory disease is one of the most important health problems in pig production worldwide. Cranioventral pulmonary consolidation (CVPC) and pleurisy are the two most common lesions in the respiratory tract of slaughtered pigs. The present review paper discusses pathogens involved in the lesions, lesion prevalence, scoring systems, advantages and disadvantages of slaughterhouse examination, and the impact of CVPC and pleurisy on performance, carcass, and meat quality. Cranioventral pulmonary consolidation and pleurisy in slaughter pigs are characteristic for infections with *Mycoplasma hyopneumoniae* and *Actinobacillus pleuropneumoniae*, respectively, although other pathogens may cause similar lesions and/or be involved in their development. The overall prevalence of CVPC and pleurisy in slaughter pigs are still high, being the prevalence of CVPC generally higher than that of chronic pleurisy. The advantages and disadvantages of slaughterhouse examination are discussed in relation to practical aspects, the assessment of lesions, the number and representativeness of the examined animals and the interpretation and value of the results for the stakeholders. The main scoring methods for CVPC and pleurisy are shortly reviewed. In general, scoring methods can be applied rapidly and easily, although significant variation due to abattoir and observer remains. Artificial intelligence-based technologies that automatically score lesions and facilitate processing of data may aid solving these problems. Cranioventral pulmonary consolidation and pleurisy have a major negative impact on pig performance, and the effects increase the extension of the lesions and/or presence of multiple lesions. The performance losses caused by these lesions, however, vary significantly between studies and farms, possibly due to differences in study population and used methodology. Both lesions also have a negative impact on different carcass and meat quality parameters, leading to increased risk for poor processing and storage of the carcasses. Monitoring lung lesions of slaughter pigs should be optimized and implemented routinely; however, it is recommended to complement this information with farm data and laboratory results for specific pathogens.

## Introduction

In the slaughterhouse, pig carcasses are evaluated by means of different parameters to assess their quality and suitability for human consumption. In addition to the food safety aspects, the slaughterhouse also represents a key check-point to capture more information about the health status of pig farms. In this sense, slaughterhouse data may complement data collected at farm level (e.g*.* clinical signs observation, necropsy findings, laboratory results and performance data) and be part of an integrated system for animal health monitoring. Lesions in many different organ systems such as the respiratory, cardiovascular, intestinal and urogenital tracts, integument and locomotion can be assessed. Such slaughterhouse data may provide unique and valuable feedback to the pig producers and their herd veterinarians, as well as be an important source of data for epidemiological and efficacy studies [1].

Evaluation of the respiratory tract is particularly important, as the porcine respiratory disease complex (PRDC) is considered the most important health problem in pig production worldwide. This is the case in intensive production systems, where large groups of pigs are housed in confinement [2]. The two most prevalent pulmonary conditions observed in pigs on abattoir inspection are suppurative bronchopneumonia, characterized by cranioventral pulmonary consolidation (CVPC), and pleuritis or pleurisy, which is most frequently seen over the caudal lobes [1, 3–5]. As these lesions are rather easy to examine at the slaughter line, they are often assessed in epidemiological studies and/or monitoring systems [1, 6]. The outcome of this assessment allows providing information about the respiratory health status of fattening pigs, to benchmark farms and to implement possible control measures against respiratory disease at the farm. The lesions are also economically important, as they are associated with increased time to market [7, 8]. In addition, they might cause problems in abattoirs because carcasses need to be trimmed due to partial condemnations [9]. This requires extra labor and may slow down the speed of the slaughter line. Finally, respiratory disease has significant negative impacts on indicators of pig welfare, as pigs may suffer from coughing, dyspnea, fever, discomfort, and reduced feed intake [10, 11].

The present review paper discusses CVPC and pleurisy lesions in fattening pigs, their characteristics, the most important pathogens involved, their occurrence, lesion scoring systems, advantages and disadvantages of slaughterhouse examination, and the impact of CVPC and pleurisy on performance, carcass, and meat quality.

## Characterization of cranioventral pulmonary consolidation and pleurisy

### Cranioventral pulmonary consolidation

In the present paper, the term cranioventral pulmonary consolidation (CVPC) will be used to describe *Mycoplasma hyopneumoniae* (*M. hyopneumoniae*)-like gross lung lesions, which consist of purple to grey areas of pulmonary consolidation, mainly located bilaterally in the apical intermediate and accessory lobes, including the cranial parts of the diaphragmatic lobes in the most extensive cases [12, 13]. In the literature, also other terms have been used to describe these lesions such as catarrhal pneumonia, bronchopneumonia, Mycoplasma-pneumonia, Mycoplasma-like lesions or enzootic pneumonia-like lesions.

Lesions caused by *M. hyopneumoniae* infections reach the maximum extension at 3–4 weeks post-inoculation [14–16]. Uncomplicated lesions are characterized by the presence of a catarrhal-purulent exudate in the airways, a uniform color of the parenchyma of the lung and a “meaty” consistency. Completely consolidated lung areas sink in water, whereas normal aerated lung areas float [11]. In incipient cases in which the consolidation does not completely affect the entire parenchyma, the lung may still float in water due to the presence of air. It takes approximately 7 to 10 weeks for the lesions to be healed, with no evidence of gross lesions anymore [12]. However, in chronic stages (more frequently seen at the slaughterhouse), consolidated areas may look purple to grey, and usually evolve to scarring and tissue retraction. These interlobular scar retractions of connective tissue are also called fissures [12, 17]. These chronic lesions are, usually, not as well coloured and demarcated as acute ones, and their scoring could be more challenging.

Under field conditions, *M. hyopneumoniae* infections are often complicated by other bacterial and/or viral pathogens [13]. In case of secondary bacterial pathogens, the lesions may occupy a larger surface of the lung and be characterized by the presence of mucopurulent exudate in the airways, a firmer tissue consistency and an inconsistent greyish color of the parenchyma. In this case, the histopathological lesion is diagnosed as suppurative bronchopneumonia.

In the case of complicating viral infections (PRDC), affected pigs show interstitial pneumonia characterized by tan-mottled, enlarged and non-collapsed lungs, rubbery in consistency, sometimes with interstitial edema. Frequently, enlargement of tracheobronchial lymph nodes does occur as well [13]. In case of complicating infections with other bacterial and/or viral pathogens, the healing, repair, and restitution periods are longer than in uncomplicated infections, and the probability of complete resolution is lower.

### Pleurisy

The serosa of the thoracic cavity, *cavum thoracicum*, is called pleura. The pleura lining the thorax is called parietal pleura and originates from the somatopleura. The pleura covering the lungs is called visceral pleura and originates from the splanchnopleura. Pleurisy, an inflammation of the pleura, is a common pathological condition observed in slaughter pigs. In the present paper, the term pleurisy will be used, although also other terms have been used in literature, such as chronic pleurisy, dorsocaudal pleurisy or pleuritis [10, 18].

Infections with *Actinobacillus pleuropneumoniae* (*A. pleuropneumoniae*) are often involved in pleurisy in pigs. In chronic cases, fibrin deposited on serosal surfaces gradually becomes organized, leading to the development of chronic fibrous adhesions between the visceral and parietal pleura. These adhesions can be firm and often result in tearing of the lungs during removal at necropsy or at the slaughterhouse, leaving portions of the lung adhered to the thoracic wall. Necrotic areas tend to resolve by fibrosis and scarring; however, in a number of cases, necrotic foci remain surrounded by scar tissue, and they are known as sequestra. Fibrous pleurisy affecting larger areas might be associated with similar lesions in the pericardial sac, namely chronic pericarditis [10]. The evolution of fibrous pleural lesions is a long process with a duration of at least one, more often two to three months [10].

## Pathogens involved in CVPC and pleurisy

Cranioventral pulmonary consolidation and pleurisy lesions in slaughter pigs are typical for infections with *M. hyopneumoniae* and *A. pleuropneumoniae*, respectively. Enøe et al. [19] demonstrated that herds seropositive for *M. hyopneumoniae* or *A. pleuropneumoniae* had CVPC and chronic pleurisy in 29% and 51% of the pigs at slaughter, respectively. In slaughter-aged pigs, the percentage of lungs with CVPC were significantly higher in *M. hyopneumoniae* seropositive farms compared to the seronegative ones [20]. *Mycoplasma hyopneumoniae* is the primary etiological agent of enzootic pneumonia (EP), a chronic respiratory disease in pigs, and it is one of the main pathogens involved in the PRDC [2, 21]. *Actinobacillus pleuropneumoniae* is a Gram-negative bacterium and the etiological agent of porcine pleuropneumonia [10]. Infections may lead to inflammation of the lung and the pleura, and may cause either acute or chronic clinical signs and lesions.

Cranioventral pulmonary consolidation and pleurisy lesions are however not pathognomonic for these two pathogens, as other pathogens may cause similar lesions and/or also be involved in these lesions as part of a polymicrobial disease complex [22]. Cranioventral pulmonary consolidation lesions can also be caused by infections with viruses, in particular swine influenza virus (IAVs) [2] and other bacterial agents such as *Pasteurella multocida* (*P. multocida*) and *Bordetella bronchiseptica* (*B. bronchiseptica*)*.* On the other hand, *A. pleuropneumoniae*, *M. hyorhinis*, *Glaesserella parasuis* (*G. parasuis*), *Trueperella pyogenes*, *streptococci* or *staphylococci* can be detected in CVPC lesions, although these lesions are not primarily caused by these pathogens [2, 23].

Pleurisy has been reported in many different studies [24–28] to be strongly associated with *A. pleuropneumoniae*. Mousing et al. [29] had calculated that 44% of all pleurisy lesions could be attributed to infection with *A. pleuropneumoniae* serotype 2. In the study of Jirawattanapong et al. [30], however, such an association was only found in 4 out of 10 farms with pleurisy. The latter authors and also others [10, 28] reported that a variety of infectious agents such as *P. multocida*, *G. parasuis* and *M. hyorhinis* can be involved in pleurisy lesions in pigs. The role of *Streptococcus suis* as causative agent of CVPC or pleuritis has not been demonstrated convincingly. In addition, observational studies found an association between the prevalence of pleurisy and the seroprevalence of pathogens that are known not to cause pleurisy, such as *M. hyopneumoniae* [19, 24, 31], porcine reproductive and respiratory syndrome virus (PRRSV) [32], porcine circovirus 2 [33] and IAVs [29, 34, 35]. These observations can probably be explained by the multi-etiological nature of the clinical respiratory problems in most farms, rather than interpreting that these pathogens can cause pleurisy. Also, progressive atrophic rhinitis was associated with pleurisy in conventional Danish pig herds, interacting with *A. pleuropneumoniae* serotype 7 [19, 29]. Paisley et al. [36] classified pleurisy lesions according to localization and indicated that pleurisy confined to the dorso-caudal lobes is primarily associated with *A. pleuropneumoniae* infections, whereas cranio-ventral pleuritis is often associated with lesions such as pericarditis and complicated pneumonia.

Wallgren et al. [28] reported that, although the prevalence of CVPC and pleurisy at slaughter can be quite similar between farms, the pathogens, as well as the infection patterns during the fattening period, can be quite variable. Also, many non-infectious parameters have been identified as risk factors for the prevalence and/or severity of CVPC and pleurisy in herds infected by bacterial pathogens able to cause these lesions [3, 6, 24, 25, 31, 37].

## Prevalence of CVPC and pleurisy lesions

Cranioventral pulmonary consolidation and pleurisy are both commonly observed lesions in slaughter pigs. The prevalence of slaughter pigs with CVPC and pleurisy lesions published in peer-reviewed international journals since 2000 are shown in Table 1. There were no published studies from North-America, possibly because lung lesion evaluation in slaughter pigs is less frequently performed in that world region.

**Table 1:** **Prevalence of cranioventral pulmonary consolidation (CVPC) and pleurisy in slaughter pigs**^**a**^

Country	Number of		Percentage of pigs with		References
	Farms	Pigs	CVPC	Pleurisy	
Australia	46	210	–^c^	15	[89]
Belgium	150 50	3,750 6,335	24 24	16 21	[3] [24]
Brazil	21 30	908 900	72 74	9 14	[31] [6]
Denmark	259 321	10 628 2064	–^c^ –^c^	27 30	[18] [19]
Finland	46	690	–^c^	6	[39]
France	1196 14 143	110 865 6997 4290	72 58 69	14 10 14	[90] [54] [25]
Germany	20	1000	73	- ^c^	[46]
Ireland	38 56	5628 23 372	58 11	43 13	[91] [34]
Italy	91 48 –^b^ –^b^	10 041 4889 216 5902	60 46 –^c^	- 48 50 42	[85] [26] [40] [48]
New Zealand	279	6 220 664	18	14	[38]
Philippines	471	1887	48	22	[92]
Serbia	20	625	42	24	[88]
Spain Spain and Portugal	107 221	10 404 199 678	56 31	27 19	[21] [17]
Switzerland	10	375	59	–^c^	[46]
Thailand	17	646	58	–^c^	[93]
UK	4420	2 261 779	27	12	[1]

^a^results from peer-reviewed studies published since 2000, and including at least 3 herds or more than 200 pigs

^b^not mentioned

^c^not investigated

The prevalence of CVPC is generally higher than that of chronic pleurisy, although that might not be the case in all countries, e.g*.* Denmark. The lowest CVPC prevalence was found in New Zealand, namely 8% [38], the highest in Brazil, around 74% [6]. The lowest pleurisy prevalence was found in Finland [39], namely 6%, and the highest in Italy, approximately 50% [40]. In a study conducted before 2000 in Denmark, Christensen and Enøe [41] showed that pleurisy accounted for approximately 70% of all post-mortem recordings at meat inspection. In a Brazilian study conducted in 30 large herds, the lesion was observed in 14% of the animals [6], while in another region of the country, in 21 farms with different production characteristics, the prevalence was 9% [31], suggesting that it is a widespread challenge.

The results show a large variation between the different studies. Part of the variation may be due to differences in study design (study population, age at slaughter), pathogen prevalence, and scoring of the lesions. The variation between individual farms and/or individual batches of slaughter pigs within each study may even be higher. Neumann et al. [38] showed that, based on data from a large national abattoir-based lesion recording system over a 10-year period, farm of origin and not abattoir, explained most of the variance in prevalence of CVPC.

Interestingly, the overall prevalence of CVPC and pleurisy in slaughter pigs since 2000 (Table 1) is similar as that reported in older studies (for review, see Maes [42]). This is surprising as some control measures against the PRDC have improved during the last two decades, such as vaccination against respiratory pathogens in fattening pigs, e.g. *M. hyopneumoniae*, PCV-2 and PRRSV. Also, much emphasis has been placed on the importance of biosecurity and tools for scoring biosecurity have been developed. The fact that the prevalence of lesions has not decreased might be attributed to suboptimal farm characteristics and insufficient implementation of proper management and biosecurity practices [39], a potential decrease in the use of antibiotics, suboptimal vaccines and vaccination practices, possible changes in the virulence and/or transmission capacity of pathogens and/or the fact that pig production has become further intensified, with fattening pigs being raised in larger groups and on larger farms. Differences in study design might also play a role. It is not clear whether the severity of the lesions has remained the same. Overall, the high prevalence values indicate that the respiratory health of fattening pigs needs to be improved, and that research focusing on improving management and biosecurity in pig farms and on developing vaccines that confer better protection is warranted. In addition to developing better control measures, also research focusing on how to increase the implementation of these measures is needed.

Lower levels of CVPC are found in countries that are almost free of *M. hyopneumoniae* such as Switzerland, Finland and Norway. In these countries, official regional and/or national eradication programs for *M. hyopneumoniae* are in place, and farms infected with *M. hyopneumoniae* are only sporadically detected [43–45]. However, and surprisingly, Luehrs et al. [46] examined the lungs of slaughter pigs from 10 Swiss pig farms that were free of *M. hyopneumoniae* and reported an overall CVPC prevalence of 59%. The lesions were mild, as their extension was less than 10% of the lung tissue for most of the pigs. This finding illustrates that the lesions observed in these farms could be caused by other bacterial pathogens different from *M. hyopneumoniae*.

The prevalence of pleurisy has traditionally been low in some North-European countries such as Finland and Sweden. However, data from recent studies [28, 39] and the Finnish Food Authority [47] showed that the prevalence of pleurisy in these countries has increased during the last years. The specific reasons are not clear, as it does not seem to be related to infection with one specific pathogen, like *A. pleuropneumoniae*. The authors of these studies [28, 39] speculate that it might result from a combination of infections with different pathogens in combination with suboptimal management and farm conditions. Most probably, the decrease of use of prophylactic antibiotics might have also contributed to this scenario. Further research is needed to explore the reasons for this increase.

## Slaughterhouse examination

Slaughterhouse examination has great potential to assess many different aspects of the respiratory health of fattening pigs such as confirmation and quantification of respiratory disease in the farm, detection of subclinical infections, and it may also serve as an important outcome parameter for various types of research studies (e.g. efficacy studies). An overview of the advantages and disadvantages of slaughterhouse examination for assessing respiratory tract lesions is shown in Table 2, which are discussed in relation to practical aspects, the assessment of lesions, the number and representativeness of the examined animals, and the interpretation and value of the results for the stakeholders.

**Table 2:** **Strengths and limitations of slaughterhouse examination for assessing the respiratory health of pigs**

	Strengths	Limitations, critical considerations
Practical aspects		
Practical arrangement with slaughterhouse	Rather easy	Sometimes access to slaughterhouse is not allowed No guarantee of precise time of slaughtering
Time needed for assessment	Short, often less than one hour	Speed of slaughter line might be too high to assess all pigs
Cost of evaluation	Inexpensive (visual assessment, palpation)	Payment might be required when removing lung plucks
Assessment of lesions		
Evaluation	Easy for experienced person	Subjective, training needed; presence of lesion (yes/no) less informative than extension of lesion
Subclinical infections	Detected in case lesions are present	Only for infections leading to gross lesions
Severity/extension of lesions	Possible	Needs more time than detection of presence (yes/no) of lesion
Detailed analysis	Possible when plucks are removed from slaughter line	Not possible at the slaughter line
Simultaneous assessment of different lesions	Possible e.g*.* pneumonia, pleurisy	Severe pleurisy may mask evaluation of other lung lesions
Examined animals		
Representative for farm situation	Easy in case all pigs of a barn are slaughtered (all-out principle)	Difficult when pigs are not marketed at once; evaluation of best or worst pigs might provide biased results
Number of animals	Large number possible (minimum 30, preferably more)	Representative sample of slaughtered batch can be selected
Interpretation of results		
Type of data	Prevalence, as lesions are examined only once (snapshot)	No incidence data (lesions not monitored over time); lesions are dynamic and can heal over time
Relevance for diagnosis	Typical lesions may suggest infection (previous or active)	No etiologic diagnosis as lesions are not pathognomonic
Benchmarking	Possible, within a farm (time evolution, barn effects), against other farms or national average	Lesions should be examined in a standardized way
Assessment of intervention strategies	Possible e.g*.* vaccinations	Lesions should be examined in a standardized way; association between lesion and carcass characteristics at animal level is not always available

### Practical aspects

Evaluation of respiratory tract lesions at slaughter is a rather easy way to collect information about the respiratory health of fattening pigs [37]. It is important to make the practical arrangements with the transport company and the slaughterhouse on beforehand. Some slaughterhouses might (temporarily) not allow access to external persons to enter the facilities and/or to evaluate the lungs e.g*.* for biosecurity reasons. Therefore, it is imperative to communicate with the person responsible for meat inspection and/or monitoring the slaughterhouse facilities.

The timing of slaughtering a specific batch of pigs cannot always be determined precisely, because of practical issues at the slaughter line or with the transport of the pigs. Therefore, the slaughtering of the pigs might be delayed or else, and even worse, when the expected arrival of other batches of pigs to the abattoir is delayed, the batch of pigs of our interest might be slaughtered earlier than the proposed time. In order not to disturb the process, it is important to understand the slaughter flowchart and to know the specific place where the evaluation will be carried out. The scoring itself can be done fast and, typically, a sufficient number of animals can be scored in a short time. An accurate assessment of lesions might be challenging in case of a fast speed of the slaughter line. In some cases, only one side of the lungs can be evaluated. In current industrial slaughter facilities, the speed of slaughtering is often more than 500 pigs per hour [48]. Therefore, a scoring system that allows quick evaluation with ease, in accordance with the speed of the slaughter line, is preferred [1]. Else, not all pigs but a subset of the population (e.g. every two or three pigs) should be evaluated.

The evaluation is also inexpensive, as it is based on visual assessment and palpation of the lungs. In case plucks are taken away from the slaughter line for subsequent more in-depth analysis, payment to the slaughterhouse might be needed.

### Assessment of lesions

Most scoring methods are based on a visual and subjective estimation of the proportion of lung affected surface and/or volume [49]. Therefore, the scoring should be done by an experienced person or after proper training. If this is not the case, it is prone to erroneous assessment. Systems for automatic recording of lesions (pictures or digital data) of the respiratory tract would not have these disadvantages [48].

Slaughter checks are also useful to detect subclinical respiratory infections, i.e*.* pigs that did not show obvious clinical signs during the fattening period, although they exhibit lung lesions. However, subclinical infections that do not lead to gross lesions of the respiratory tract are not detected [50].

The presence as well as the severity of different types of lung lesions, considering the resolution time of such lesions, can be assessed. Detailed analysis or simultaneous assessment of many different lesions might be difficult or impossible in slaughterhouses with high speed of the slaughter process. In this case, one should focus on a sample of pigs and/or remove the materials from the slaughter line (when allowed) and transfer them to an appropriate location for more detailed assessment.

Another disadvantage is that the presence of severe pleurisy may obscure the evaluation of CVPC [49]. This is especially the case when major parts of the lung stick to the inner chest wall of the carcasses.

### Examined animals

It is important that the examined pigs are representative for the situation on the farm. Assessment of the best or worst performing pigs of a batch will lead to biased results. This risk is higher in case the pigs of a barn are not sent to slaughter at once, but in two or three successive times over a few weeks. In such scenarios, the first pigs are usually those with the highest growth and health status, and therefore, have suffered the least from respiratory disease. The opposite might be the case for the smallest pigs of a group. These pigs might have had more health problems, and therefore, can result in an overestimation of the prevalence of lung lesions at the farm.

Usually, not all pigs but a subset of representative lungs from the farm is examined. Although a minimum of 30 randomly selected pigs from the same slaughter batch has been shown to provide reliable information at herd level [51], it is generally accepted that it is better to evaluate more pigs, or to make the sample proportional to the size of the batch. Statistical criteria to obtain the preferred accuracy for prevalence and/or severity scoring should be used. To this end, the size of the population at risk, the expected prevalence, and the desired confidence in the probability of detecting disease in the sample should be considered. The pigs to be examined can be selected by different sampling strategies, such as random sampling or systematic sampling, in which one pig every e.g*.* two, three or four pigs is scored.

### Interpretation of the results

Examination of the respiratory tract at the slaughterhouse generates prevalence data, as pigs are not monitored over time, but examined only once. However, lung lesions are dynamic, implying that lesions in young animals may have healed at the time of slaughter. This regression of lesions during the fattening period may therefore lead to false-negative results [52]. Pessoa et al. [53] performed an observational study in which groups of pigs were monitored weekly during the entire fattening period and at slaughter. They found positive associations between the prevalence of CVPC at slaughter and coughing during the last three weeks of the fattening period, but not with coughing earlier during the fattening period. They also found positive associations between the prevalence of fissures (attributed to chronic *M. hyopneumoniae* lesions) and coughing 7 to 3 weeks prior to slaughter. This suggests that lesions at slaughter reflect the respiratory health of the pigs during the last weeks of the fattening period, but that they are a poor indicator of the respiratory health of the pigs in the early fattening phase. This corroborates findings of Pagot et al. [54] and Baraldi et al. [31]. The healing of the lesions and the fact that some subclinical infections are not detected, may lead to an underestimation of the respiratory problems when relying solely on slaughterhouse examination. On the other hand, some infections leading to lesions may take place at the end of the fattening period only. In this case, a high prevalence and/or severity of lesions may be observed at slaughter, whereas the pigs suffered from the health problem only for a short time, at the end of the fattening period. In this case, slaughterhouse evaluation results might overestimate the problems encountered during the fattening phase. Also, when very small lesions are counted, prevalence data might lead to an overestimation of the importance of the problem.

Apart from the dynamic nature of lung lesions, it is also important to realize that lesions might be typical, but that most lesions are not pathognomonic, that is not being specific for a pathogen. Therefore, slaughterhouse examination does not allow establishing an etiologic diagnosis. The results should be regarded to complement clinical and performance data from the farm and results from necropsy and laboratory examinations.

Slaughterhouse data can be used to investigate differences in the respiratory health of pigs raised in different barns within a farm, to assess evolution over time, and to benchmark the farm against other groups of farms or the national average. In this sense, slaughter data important in health schemes and for increasing awareness of these lesions by farmers [1]. Also, the effectiveness of intervention strategies can be evaluated. For efficacy trials or for assessing the impact of lesions on performance, the link between lesion and carcass characteristics is needed at animal level. Depending on the situation in the abattoir, it might not be possible to obtain this information.

## Scoring systems for CVPC and pleurisy

**Figure 1:**
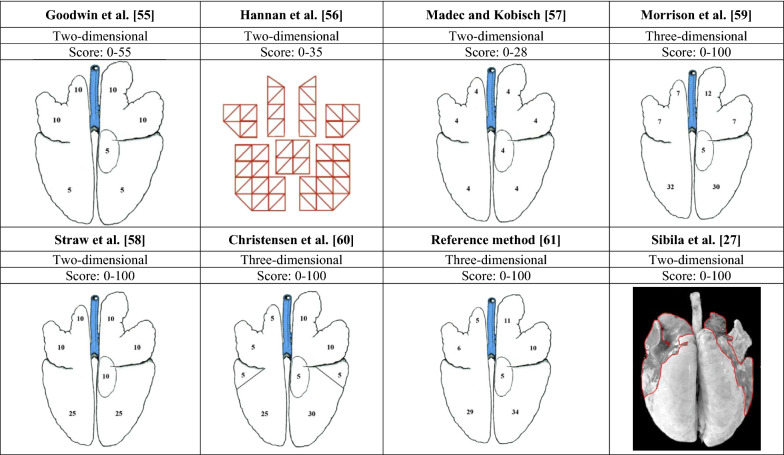
**Commonly used methods to score cranioventral pulmonary consolidation in slaughter pigs (adapted from **[49]**).**

Different lung scoring systems have been developed over the past decades (Figure 1, Table 3). They are based on visual and manual assessment of the affected proportion of the lung [49]. Most of the scoring systems use a two-dimensional approach by assessing the percentage of lung surface that is affected [27, 55–58]. Other methods use the percentage of affected lung volume or weight and are therefore based on the three-dimensional characteristics of the lung [59–61].

**Table 3:** **Overview of pleurisy scoring systems in slaughter pigs**^**a**^

Score	Scoring system				
	Madec and Kobisch [57]	CTPA ^b^	Pointon et al. [66]		SPES ^c^
0	No pleurisy				
1	Interlobular pleurisy (visceral pleurisy)	Fibrinous pleurisy	Interlobular pleurisy (adhesions between lung lobes)	1 N: pleurisy with normal lungs	Cranioventral lesion: interlobar pleurisy or at ventral border of caudal lobes
				1P: pleurisy with pneumonic lungs	
2	Localized pleurisy < 2 cm diameter	Extended pleurisy: lungs cannot be removed from the carcass	Pleurisy (adhesions of lungs to chest wall)	2 N: pleurisy with normal lungs	Dorsocaudal monolateral focal lesion
				2P: pleurisy with pneumonic lungs	
3	Extensive pleurisy > 2 cm diameter with adhesions to ribcage	–	–		Bilateral lesion of type 2 or extended monolateral lesion (at least one third of one diaphragmatic lobe)
4	Partial or total ribcage condemnation	–	–		Severely extended bilateral lesion (at least one third of both diaphragmatic lobes)

^a^The Danish system [41] is explained in the text but not listed in the table, as it differs significantly from the other systems

^b^CTPA System by the Centre Technique de Productions Animales[54]

^c^SPES Slaughterhouse Pleurisy Evaluation System[67]

Some slaughter‐check diagrams include more detailed information about the lung, such as the type of pneumonia, location, the presence of other lesions on the lung (e.g. pleurisy, adhesions, abscesses) and/or evaluations of the liver and heart [60]. In some cases, drawing the lesion may be of help to better calculate the final scoring. Nevertheless, if this option is not feasible (due to the speed of the chain), an effective alternative to drawings would be digital images or photographs of lungs [62]. These images can/need to be reviewed later, which is time-consuming and slaughterhouses may limit the use of a camera in the premises. Using a voice activated recording device greatly simplifies records and allows the hands to manipulate the pluck.

### Scoring of CVPC

An overview of commonly used scoring methods for CVPC in pigs has been published by Garcia-Morante et al. [49] (Figure 1). They include the system described by Goodwin et al. [55], Hannan et al. [56], Madec and Kobisch [57], Morrison et al. [59], Christensen et al. [60], Sibila et al. [27], Straw et al. [58] and the system recommended by the European Pharmacopoeia (Ph. Eur., monograph number 04/2013:2448) [61] for the evaluation of porcine enzootic pneumonia as reference system. Other systems have also been described, e.g. Steinmann et al. [63], but they are not further discussed as frequently they derive from one (e.g. [58]) of the eight systems of Figure 1. The affected lung tissue score can be expressed as a number [55–57], or as a percentage [59–61]. Garcia-Morante et al. [49] reported high correlations between the different scoring systems. Also, Sitjar et al. [64] had found two measurement techniques based on either superficial or volumetric pneumonia to provide essentially identical information.

Some scoring systems have been developed to assess CVPC in pigs under experimental conditions when there is more time for the assessment [56], whereas others are more suitable for a rather fast assessment of the lesions under field conditions [59]. The scoring system described by Hannan et al. [56] is appropriate to carefully quantify lung lesions caused by experimental infection with *M. hyopneumoniae*. The score ranges from 0 (no lesions) to 35 (all lung tissue affected). The lesions are first sketched onto a standard diagram of the lung, followed by analysis of sketches on the diagrams to determine the overall lung score.

### Scoring of pleurisy

An overview of commonly used scoring systems for pleurisy in slaughter pigs is summarized in Table 3.

#### System by Madec and Kobisch

The first scoring system for pleurisy was developed by Madec and Kobisch [57]. This scoring system has the advantage of defining a score 4 for advanced stages likely having a major effect on performance and/or leading to carcass condemnations. Nevertheless, it should be ensured that non-evisceration is due to pleural adhesions and not to incorrect carcass preparation. Interlobar pleurisy, score 1, is easily identified by handling the lungs. However, localized pleurisy with a diameter smaller than 2 cm, score 2, requires good attention and may only be visible in good light. Sensitivity of scoring mild pleurisy cases may vary more between assessors [65] and emphasizes the need for training. Extensive pleurisy, score 3, and partial or total ribcage condemnations, score 4, are obvious and in most cases do not need palpation, but require carcass inspection.

#### System by the centre technique de productions animales (CTPA)

The Centre Technique des Productions Animales (CTPA) in France uses a simplified scoring system from 0 to 2 for on-farm trials [54]. It is a simple system for clinical trials with score 0 for absence of pleurisy, score 1 for fibrinous pleurisy and score 2 for extended pleurisy with adhesions to the ribcage. However, as stated in the original paper, score 1 would apply exclusively to acute forms of pleurisy (presence of fibrin) and does not apparently include the possibility of mild chronic forms with minimal adhesions to the thoracic cavity. In contrast, score 2 indicates that lungs cannot be easily removed from the carcass, which indicates exclusively chronic lesions with marked adherences. Therefore, a significant number of lungs found at abattoir might have lesions that should be considered in between those scoring values and, in consequence, certain classification difficulties may arise.

#### System by Pointon et al. [66]

In North America and Australia, a system is used that specifically differentiates pleurisies with adhesions between lobe lungs (score 1) or between lungs and wall chest (score 2) [66]. Moreover, the scores take into account whether or not the pleurisy is associated with pneumonia (N: pleurisy without pneumonia; P: pleurisy with pneumonia). The scoring system is used in the American (PigMON) and Australian (PHMS, Pig Health Monitoring Schemes) monitoring schemes.

#### Slaughterhouse pleurisy evaluation system (SPES)

The Zootechnical Institute of Lombardy and Emilia-Romagna (Italy) devised a new scoring system entitled SPES (Slaughterhouse Pleuritis Evaluation System) [67]. The system aims to determine the extension and localization of the lesions by focusing on the diaphragmatic lobes. It has also been used in other studies [21, 24, 27]. Di Provvido et al. [40] investigated an alternative method for pleurisy scoring, namely the PEPP (pleurisy evaluation of the parietal pleura). The method is easy to conduct and involves the evaluation of the parietal pleura i.e. the serous membrane lining the chest wall. Very high correlations were found with the SPES method.

#### System by Christensen et al. [41]

The Danish system of Christensen et al. [60], which is not listed in the table due to its dissimilarity with the other systems, involves careful examination of the lungs and sketching of the lesions in a record form [60]. It is much more precise than other systems, but it is not suitable for use at the slaughter line. The score is proportional to the damaged surface area on each lobe and for each side of the lungs (cranioventral and dorsocaudal).

#### System by Sibila et al. [27]

This system, developed in Spain, is based on the quantification of damaged lung surface by delimitating, in a picture and using an image software, the area of the affected lung tissue [27]. This system requires to take pictures from both sides of the lung (if presence of lesions at the ventral side is confirmed) and the analyses of the images through a software, indicating the percentage of affected area. Therefore, this system is difficult to be performed at the slaughterhouse. It has been used to analyze the outcome of experimental infections with *A. pleuropneumoniae* [27] as well as *M. hyopneumoniae* [49].

### Artificial intelligence to score lesions

The mentioned scoring systems are generally simple and easy to standardize. However, they are rather time-consuming, and abattoir-related and inter-observer variations cannot be fully ruled out. Artificial intelligence-based technologies are under development to automatically score pictures of pleurisy in slaughtered pigs [48]. The automation of such a process would be extremely helpful to enable a systematic examination of all slaughtered livestock [68]. In the study of Trachtman et al. [48], artificial intelligence was able to differentiate pleural surfaces affected with pleurisy from healthy ones, with an overall accuracy of 86%. The system was better able to make distinction between severely and moderately affected pleural surfaces as compared to evaluation by experienced veterinarians. Further research is needed to train the neural networks to identify and score pneumonia, and to implement the system in large capacity slaughterhouses. The technology could provide the data in a suitable form for statistical analysis, thereby facilitating data processing and the supply of summaries and recommendations to industry stakeholders. Nevertheless, these systems will depend, among other factors, on the quality and precision of the pictures. The latter issue is not easy to be solved in a non-static hanged pluck of lungs.

## Impact of CVPC and pleurisy on performance

Various approaches have been used to estimate productivity losses and economic impact of respiratory disease under field conditions. A commonly used strategy is to compare the performance of pigs showing lesions of the respiratory tract at slaughter with the performance of pigs not showing lesions, or else to compare the performance of batches of pigs with different prevalence of CVPC or pleurisy. Studies that have investigated the impact of CVPC and pleurisy in slaughter pigs are discussed below.

### Cranioventral pulmonary consolidation

#### Impact of presence of CVPC

Morrison et al. [59] studied 23 publications to examine the possible negative effect of CVPC at slaughter on average daily gain (ADG). In 10 studies, no negative effect of CVPC was identified; in the other studies, a negative association was found. Pagot et al. [54] reported that the ADG during the fattening period was approximately three percent lower for pigs showing CVPC at slaughter. They detected large differences between farms; in some farms, the ADG decrease in pigs with CVPC reached up to 7%. Also, Paisley et al. [36] showed substantial variation between herds. The presence of CVPC in two farms was associated with a decreased ADG during the fattening period of 31 and 137 g respectively. In a recent study in one farm, Paz-Sánchez et al. [8] found that the presence of CVPC reduced ADG by 9%.

Martínez et al. [69] did not find a negative effect of CVPC lesions on ADG and feed efficiency. In that study, performance data were analysed at farm level, whereas lung lesions were assessed at slaughter from one subgroup (35 pigs) per farm only. The subgroup might not have been fully representative for the farm and the number of farms might have been too small to observe statistical differences.

#### Impact of severity (extension) of CVPC

Several studies also examined the effect of severity of CVPC on performance (Table 4). The term severity is used here to refer to the extension of the pneumonic lesion. Straw et al. [51] calculated, based on the results of five studies, that ADG is decreased by 37.4 g for every 10% of the pig’s lung surface affected by CVPC. They also demonstrated that, on average, CVPC caused 17% and 14% decreases in ADG and in feed efficiency, respectively. The same authors further reported that the percentage increase in feed conversion ratio (FCR) was highly correlated with the percentage decrease in ADG, meaning that when there is a negative effect on ADG, one can also expect a negative effect on FCR, and vice versa.

**Table 4:** **Studies assessing the impact of cranioventral pulmonary consolidation (CVPC) on average daily gain (ADG) in pigs**.

References	Study population	Impact on ADG	Comment
**Presence of CVPC**			
[59]	23 studies	10 publications: no effect 13 publications: decrease	
[51]	9 studies	−17%	Feed conversion ratio +14% High correlation between decreased ADG and feed conversion ratio
[36]	2 farms (578 pigs)	Farm A: −26 g Farm B: −26 g	
[54]	14 farms (6,997 pigs)	−38 g (−4.9%)	Large differences between farms (range: −7% to +2.6%
[69]	39 farms	No effect	No effect on feed conversion ratio Performance data were analysed at farm level; lung lesions were assessed on one subgroup (35 pigs per farm) at slaughter
[8]	1 farm (108 pigs)	−9%	
**Severity of CVPC**			
[51]	5 studies	37.4 g per 10% of affected lung tissue	Five studies
[52]	21 pigs	20% CVPC throughout lifetime: 25 kg lower weight at slaughter	Radiographic monitoring of the lungs from 21 to 180 days of age 25 days extra to reach slaughter weight
[7]	2 farms (41 lungs)	41.1 g/day for 10% affected lung volume	10% affected lung leads to 16.7 days more to reach 104.5 kg
[70]	2 farms (58 lungs)	31.4 g/day for 10% affected lung volume	10% affected lung leads to 13·2-days more to reach 104.2 kg Only significant association between lung lesion and ADG in one farm
[72]	1 farm (333 pigs)	2.2 g/day for each 1% of affected lung volume	1% affected lung leads to 0.61 days more to reach slaughter weight; early infections cause more performance loss
[50]	7 farms (18 cohorts with 30–35 pigs / cohort)	6.8 g/day for 1% of affected lung	Serologic testing was more effective than slaughter evaluation in assessing the impact of subclinical infection on ADG
[73]	1 farm (138 pigs)	Score 2: −45 g/day (−6%) Score 3: −90 g/day (−11%)	Lung consolidation: score 1: 0–5%; score 2: 6–40%; score 3: > 40% Score 2: 3.13 kg less at slaughter Score 2: 10.85 kg less at slaughter
[54]	14 farms (6,997 pigs)	−7.0 g/day (0.7%) for 1 point increase in severity score (1–28)	More ADG decrease in pigs with severe lesions: score 0: 775 g; scores 1–4: 750 g; scores 5–8: 735 g; score > 8: 695 g
[71]	1 farm (500 pigs)	1.8 g/day for each 1% of affected lung surface	Financial loss of §6.55 in pigs with more than 15% affected lung tissue compared to animals without lesions
[8]	1 farm (108 pigs)	−66.4 g/day in pigs with > 10% of affected lung surface versus pigs with no lesions:	Days to slaughter: + 8 days; carcass weight: −3.6 kg

Hill et al. [7] indicated that a 10% increase in the volume of pneumonic tissue (within the range of 1 to 70% affected lung volume) was associated with a decrease in ADG by 41.1 g, and a 16.7-day increase in number of days to reach 104.5 kg. Very similar results (−31.5 g ADG decrease for each 10% of affected lung volume) were obtained in a follow-up study two years later [70]. Regula et al. [50] found a decrease in ADG of 6.8 g for each percent of affected lung tissue. A recent study [71], obtained results that were in between the two previous studies, namely 1.8 g decrease in ADG during the fattening period for one percent increase in area lung consolidation, corresponding with 18 g decrease in ADG for 10 percent affected lung tissue. Similar results were obtained by Christensen [72], who showed that each 1% of lung volume affected by CVPC lesions reduced DWG by 2.2 g, and increased the number of days to reach slaughter weight by 0.61 days. He further calculated that the growth-depressing effects of *M. hyopneumoniae* infections are, on a relative scale, more pronounced in pigs with severe CVPC. Donkó et al. [73] used the scoring method of Straw et al. [58] and showed a reduction in ADG during the fattening period of 5.5% and 11% for pigs with CVPC score 2 (5–40% of lung tissue consolidated) and score 3 (>40% of lung tissue consolidated), respectively.

Pagot et al. [54] reported that the CVPC score at slaughter explained 19% of the ADG variation. One supplementary point in the CVPC Madec score (0–28) corresponded with approximately 0.7% of lost growth.

Other studies emphasized the importance of the dynamic nature of lesions and/or time of infection when assessing the impact on performance. Noyes et al. [52] and Wallgren et al. [74] indicated that CVPC lesions present at slaughter are a poor indicator of lifetime pneumonia, as lesions can progress and regress throughout the life of pigs. They indicated that the approximate time of infection should be included to obtain a useful interpretation of the severity and impact of CVPC lesions at slaughter. Morris et al. [75] and Sitjar et al. [64] showed that weight losses due to *M. hyopneumoniae* infections are more substantial when pigs are infected early in life, as they have suffered from the respiratory problems for a longer time.

Paisley et al. [36] showed that the presence of interlobular fissures or scars at slaughter, which are considered to be old or healed CVPC lesions [12], was associated with a reduced ADG early in the fattening period, while CVPC (recent Mycoplasma-like lesions) at slaughter was associated with a reduced ADG in the period close to the time of slaughter. Noyes et al. [52] calculated that a pig with 20% affected lung tissue throughout its lifetime, measured by radiographic examinations throughout the fattening period, weighs 25 kg less or reaches slaughter weight about 25 days later than a pig without CVPC. In a later study, Sitjar et al. [64] did not find an overall significant association between lifetime pneumonia and reduced ADG. They indicated that the discrepancy with the study of Noyes et al. [52] might be due to the fact that the farm studied had a less severe respiratory problem than the one studied previously. Even so, Sitjar et al. [64] did find a strong correlation between average lifetime pneumonia and decreased ADG in lightweight pigs, which generally had more severe CVPC than heavier weight animals.

Other studies did not only focus on the presence or severity of the lesions, but also emphasized the importance of lesions being the result of either a single infection or mixed infection, and therefore included different disease parameters to assess the impact on performance. The effect of a single *M. hyopneumoniae* infection on ADG and FCR can only be assessed under experimental conditions, since almost all field infections are complicated by other infectious agents. The effects of single *M. hyopneumoniae* infections on ADG are variable, with some studies [76, 77] showing pronounced negative effects on ADG (−117 g/d and −10–20%, respectively), whereas other studies did not find a negative effect [49, 78]. The different results might be explained by differences in the challenge model or other aspects of the study design. Experimental challenge involves a limited number of animals, implying that the statistical power is often (extremely) low to show significant differences in parameters with a large variation such as ADG. Also, a small proportion of dead pigs might have a large impact on the outcome. Furthermore, challenge models in which high doses of *M. hyopneumoniae* organisms are inoculated once or twice and in which pigs are monitored for a short period (after infection), are not reliable to mirror the field situation of a chronic respiratory disease. Therefore, challenge studies are not very suited to assess the impact on performance.

Rautiainen et al. [43] compared the performance of individual pigs during the fattening period of two farms based on their serological status against *M. hyopneumoniae* and CVPC at slaughter. Pigs with *M. hyopneumoniae* serum antibodies and CVPC at slaughter, grew from 75 to 176 g/day slower (10.2% to 17.5% reduction) than pigs without antibodies and CVPC. They also showed that pigs that became seropositive for *M. hyopneumoniae* late during the fattening period without or with only very mild CVPC had a reduced ADG (60 g/day) compared to seronegative pigs without CVPC [43]. Similarly, Regula et al. [50] reported that *M. hyopneumoniae* seropositive pigs had lower ADG (38 g/day) compared to those that were seronegative. The latter authors suggested that regular monitoring for antibodies against respiratory pathogens during the fattening phase was more effective in assessing the impact of subclinical infection on performance than monitoring lung lesions at slaughter.

### Pleurisy

Pagot et al*.* [54] reported that pleurisy at slaughter was associated with an ADG decrease of 39 g (5.2%), and a 6-day increase in the length of the fattening period (Table 5). They also found that CVPC and pleurisy had a synergistic effect. Hence, animals showing CVPC and pleurisy suffered from 15% lower growth than non-affected pigs.

**Table 5:** **Studies assessing the impact of the presence of pleurisy on average daily gain (ADG) in pigs.**

Reference	Study population	Impact on ADG	Comment
[79]	9592 pigs from 78 farms; examined in 4 slaughterhouses		Fattening period + 8 days
[51]	4 studies	−34%	Feed conversion ratio + 26%
[29]	4800 pigs, selected on specific days in one slaughterhouse	Lower carcass weight	Effect was shown in multivariable logistic model with carcass weight being negatively associated with pleurisy lesions
[36]	2 farms (578 pigs)	Farm A: no effect Farm B: -28 g /−42 g	Effects in farm B were depending on localization of pleurisy: dorsocaudal pleurisy: no effect; parietal pleurisy: −28 g; antero-ventral pleurisy: −42 g
[54]	14 farms (6997 pigs)	−39 g (−5.2%)	Large differences between farms (range: -19.9% to +4.6%) Severity of pleurisy: score 1: −54 g; score 2: −37 g Fattening duration +6 days Pleurisy and CVPC: ADG −15% (synergistic effect)
[69]	39 farms (one batch per farm)	No effect	No effect on feed conversion ratio Performance data were analysed at farm level; lung lesions were assessed on one subgroup (35 pigs) at slaughter
[8]	1 farm (108 pigs)	−11%	

Straw et al. [51] demonstrated that, on average, pleurisy decreased ADG and FCR by 34% and 26%, respectively. Paz-Sánchez et al. [8] found an ADG decrease of 11% in pigs with pleurisy. Mousing et al. [29] showed that pigs with pleurisy had a lower carcass weight at slaughter, and Hartley et al. [79] showed that pleurisy was associated with increased time (up to 8 days) to slaughter.

On the contrary, Paisley et al. [36] showed that pleurisy without the presence of other lesions was not associated with decreased performance. Also, Martínez et al. [69] could not observe a negative effect of pleurisy on performance. As mentioned before, some limitations of the latter study might have resulted in lack of identifying a potential relationship.

Jäger et al. [80] estimated the cost of pleurisy in British pig herds to be £5 (approximately €6) per pig produced. Their calculations were based on industry standard costs associated with 6% increase in mortality during the grower-finisher period, 20% prevalence of pleurisy, 50 g reduction in ADG and 0.1 reduction of FCR. Ferraz et al. [71] estimated a cost of US$6.6 (approximately €6.4) per pig produced considering a reduction in ADG of 27 g associated with lesions (CVPC and pleurisy) in ≥ 15% of the lungs compared with pigs without lesions. Calderón Díaz et al. [81] showed, using a bio-economic simulation study, that from six recorded pluck lesions, pleurisy and lung scars were the main lesions associated with decreases in ADG during the grow-finisher period and a lower economic return.

Apart from a decrease in average performance, there is a considerable cost associated with unequal rates of growth of pigs within a herd. Slow growing pigs must be held back and marketed later, or the producer must accept a deduction for lightweight pigs at slaughter. These hidden costs must be considered when calculating the costs and benefits of intervention strategies.

In some cases, pigs may have more than one type of lesion in the respiratory tract or in other organs, e.g. the liver [1]. In that case, such lesions may also influence the performance of the animals. This can lead to comparison of growth rates in groups of pigs with different diseases or combinations of diseases, rather than comparing diseased and non-diseased groups. This might result in over- or under-estimation of the importance of some lesions.

In addition to the performance losses for the farmer, pleurisy also incurs losses for the abattoirs because pleurisy requires trimming of the carcass causing disruption, reducing line speed, and increasing labor and wastage costs. Some processors might no longer want to bear these costs, implying that producers that keep submitting consignments with high pleurisy prevalence might be penalized in the future.

## Impact of CVPC and pleurisy on carcass and meat quality

Pigs affected with lung lesions eat less, and therefore, they do not fully use their genetic potential for muscle synthesis and fat accumulation [82, 83]. Paz-Sánchez et al. [8] observed that pigs without lung lesions had an average carcass weight of 77.7 kg at 200.8 days, whereas the carcass weight of pigs with lung disease was 75.5 kg at 206 days old. In a first study, Escobar et al. [78] reported that a single experimental *M. hyopneumoniae* infection did not affect whole-body fat or protein accretion of nursery pigs during a four-week experiment. In a later study using experimental infection with *M. hyopneumoniae* and PRRSV [84], they reported that the magnitude of increases in inflammatory cytokines is predictive for decreases in protein accretion and growth. The authors further suggested that during infection, growth of skeletal muscle is limited in part by increased levels of myostatin, a circulating factor that may act as a muscle chalone.

Ostanello et al. [85] investigated 109 batches of Italian pigs of approximately 160 kg (9–10 months of age). They found a significant negative association between the mean CVPC score of a batch of slaughter pigs and the carcass quality, based on the EUROP evaluation grid. That system uses percentage of lean meat content of the carcasses, batch homogeny and carcass attitude for the production of raw ham as carcass quality parameters. The authors speculated that the negative effects on carcass quality might be due to hypoxia due to the pulmonary lesions.

Lung lesions may also cause changes in pH, water holding capacity, color, flavor, and cooking quality loss of the meat [86]. Permentier et al. [87] showed that the pH values measured at the end of the slaughter line in the loin of pigs with more than 25% and less than 5% lung lesions, were 6.01 and 6.20, respectively. This indicates pigs with lung lesions lead to a higher risk for pale, soft and exudative (PSE) traits and quality losses in cooked or dry-cured ham. The other meat quality parameters were not significantly different between pigs with and without lung lesions.

Čobanović et al. [88] investigated 625 pigs originating from 20 small-scale farms and assessed the influence of different lung lesions on carcass and meat quality parameters. Pigs with CVPC and pleurisy had the lowest live weight, hot and cold carcass weight and meatiness. Additionally, pigs with multiple lesions had lower live and carcass weights than pigs affected by a single pathological condition. Likely, this is due to repartitioning of nutrients from muscle deposition and bone growth towards tissues where repair processes are needed (e.g. lung tissue affected by pneumonia).

In a study of Karabasil et al. [83], pigs with CVPC (pH 6.23) and pleurisy (pH 6.23) had higher average pH in the carcasses at the end of the slaughter line compared to pigs without lesions (pH 6.14). The percentages of carcasses with PSE as well as with DFD (dry, firm and dark) meat were significantly higher in pigs with CVPC (PSE 9.7%; DFD 12.6%) or pleurisy (PSE 8.0%; DFD 16.0%) compared to pigs without lesions (PSE 3.9%; DFD 5.1%). This results in meat with a higher risk for poor processing characteristics, bacterial growth and a reduced shelf life. Similar findings had been obtained in an earlier small-scale (79 pigs) study [83].

## Conclusions and areas for further research

Cranioventral pulmonary consolidation and pleurisy lesions in slaughter pigs are typical for infections with *M. hyopneumoniae* and *A. pleuropneumoniae*, respectively, but the lesions are not pathognomonic, and other pathogens may cause similar lesions and/or also contribute to these lesions. To have a better view of the pathogens involved, additional diagnostic testing for infectious agents and their infection patterns should therefore be conducted. Attention should be paid to the possible polymicrobial nature of the lesions, the effects of possible immunosuppressive pathogens and the environmental conditions on the farm.

The overall prevalence of CVPC and pleurisy in slaughter pigs is high and similar to values reported two to four decades ago. Possibly, the extension of the lesions has decreased in the last decades. There is also a large variation between the different studies, and among individual farms. This implies that future research should be directed towards the development and implementation of better control measures such as vaccination strategies, management practices, biosecurity, and biocontainment.

Evaluation of CVPC and pleurisy at slaughter is simple, fast, and rather easy to conduct, but variation due to abattoir, observer and method might be an issue. Artificial intelligence-based technologies hold great promise to automatically score lesions and facilitate useful feedback to the farmer, the herd veterinarian and other stakeholders. More research is needed to test and optimize this technology, to develop it for different (lung) lesions in slaughter pigs and to investigate the feasibility of its reliable implementation in different slaughterhouse conditions.

Cranioventral pulmonary consolidation and pleurisy in batches of slaughter pigs have a negative impact on performance of the pigs, and the negative effects are more pronounced with the prevalence and the severity of the lesions. The results however vary between studies and farms. This is likely due to differences in study design (type of pathogens involved, infection levels, infection patterns, farm characteristics and management, age of slaughtering) and methods used to assess the lesions. The major economic losses due to these lesions suggest that research in developing better control measures is warranted and will pay off for the farmer and the entire pig sector. In addition to the losses for the farmer, CVPC and pleurisy also decrease carcass and meat quality. The economic losses in this part of the pork production chain have largely been neglected so far, but they can be tremendous as there is an influence on food quality and safety, and the shelf life of the carcasses. Research in quantifying these economic losses and the impact for food safety and public health is needed.

Given the high prevalence and impact of CVPC and pleurisy lesions for the farmer, the slaughterhouse and all stakeholders of the pig sector, monitoring lung lesions of slaughter pigs should be optimized and be implemented routinely for as many farms as possible. It is recommended to complement this information with other farm data such as clinical scores, antimicrobial use, animal behavior and welfare scores and performance data to obtain a more holistic view of the respiratory health status of the farm.

## Abbreviations

*A. pleuropneumoniae*: *Actinobacillus pleuropneumoniae*
ADG: average daily gain
*B. bronchiseptica*: *Bordetella bronchiseptica*
CTPA: centre technique des productions animales
CVPC: cranioventral pulmonary consolidation
DFD: dry, firm and dark
EP: enzootic pneumonia
FCR: feed conversion ratio
*G. parasuis*: *Glaesserella parasuis*
*M. hyopneumoniae*: *Mycoplasma hyopneumoniae*
*M. hyorhinis*: *Mycoplasma hyorhinis*
PCV2: porcine circovirus 2
PEPP: pleurisy evaluation of the parietal pleura
PRDC: porcine respiratory disease complex
PRRSV: porcine reproductive and respiratory syndrome virus
PSE: pale, soft and exudative
SPES: slaughterhouse pleurisy evaluation system
